# Supramolecular Organization in Salts of Riluzole with Dihydroxybenzoic Acids—The Key Role of the Mutual Arrangement of OH Groups

**DOI:** 10.3390/pharmaceutics15030878

**Published:** 2023-03-08

**Authors:** Alexander P. Voronin, Artem O. Surov, Andrei V. Churakov, Mikhail V. Vener

**Affiliations:** 1 G.A. Krestov Institute of Solution Chemistry RAS, 153045 Ivanovo, Russia; 2 Kurnakov Institute of General and Inorganic Chemistry, Russian Academy of Sciences, Leninskii prosp. 31, 119991 Moscow, Russia

**Keywords:** riluzole, hydroxyl-derivatives of salicylic acid, periodic DFT calculations, enthalpy of intermolecular H-bonds, 1D and 2D hydrogen bond networks, the effect of positional isomerism on the supramolecular organization

## Abstract

Intermolecular interactions, in particular hydrogen bonds, play a key role in crystal engineering. The ability to form hydrogen bonds of various types and strengths causes competition between supramolecular synthons in pharmaceutical multicomponent crystals. In this work, we investigate the influence of positional isomerism on the packing arrangements and the network of hydrogen bonds in multicomponent crystals of the drug riluzole with hydroxyl derivatives of salicylic acid. The supramolecular organization of the riluzole salt containing 2,6-dihydroxybenzoic acid differs from that of the solid forms with 2,4- and 2,5-dihydroxybenzoic acids. Because the second OH group is not at position 6 in the latter crystals, intermolecular charge-assisted hydrogen bonds are formed. According to periodic DFT calculations, the enthalpy of these H-bonds exceeds 30 kJ·mol^−1^. The positional isomerism appears to have little effect on the enthalpy of the primary supramolecular synthon (65–70 kJ·mol^−1^), but it does result in the formation of a two-dimensional network of hydrogen bonds and an increase in the overall lattice energy. According to the results of the present study, 2,6-dihydroxybenzoic acid can be treated as a promising counterion for the design of pharmaceutical multicomponent crystals.

## 1. Introduction

The design of pharmaceutical multicomponent solids, primarily relies on the utilization of supramolecular synthons, as reliable building blocks that enable the construction of molecular assemblies with desired structural features and properties in a predictable manner [1,2,3]. Even though various types of intermolecular interactions concurrently exist in the crystal structure and contribute to its thermodynamic stability [4,5,6,7], intermolecular hydrogen bonds (H-bonds) play a more prominent role than others, due to their strength and directionality [8], allowing crystal engineering principals to be used [9,10,11,12]. However, the possibility to properly regulate or anticipate the formation of multiple supramolecular synthons in multicomponent crystals, consisting of molecules with a diverse spectrum of functional groups, remains a challenge, due to a lack of understanding of the nature and strength of intermolecular interactions responsible for competition between different synthons in organic solids [13,14,15]. Therefore, it is of great importance to provide a quantitative evaluation of the binding strength and nature of synthons, to rationalize their stabilizing and structure-directing roles in the formation of multicomponent crystals [16,17,18,19,20,21].

Riluzole (2-amino-6-trifluoromethoxy-benzothiazol) is one of the few approved options to treat amyotrophic lateral sclerosis [22]. Treatment with riluzole (RLZ) leads to slower degradation of neurons and extended survival without tracheostomy [23,24]. The mechanism of action of riluzole is complex, involving glutamatergic neurotransmission enhancement [25], inhibition of fast and persistent sodium currents [26,27], neuroprotective activity [28], as well as neuroplasticity stimulation via stimulation of the neurotrophic factors [29,30]. RLZ was shown to exhibit neuroprotective, anticonvulsant, and antidepressant activity in multiple in vivo studies [31]. In addition, RLZ can be used for the treatment of severe mood, anxiety and impulsive disorders, including obsessive-compulsive disorder, unipolar and bipolar depression, and generalized anxiety disorder [32,33]. Recent studies have demonstrated the potential of repurposing RLZ as an antiproliferative agent for many types of cancer, including skin, breast, pancreas, colon, liver, bone, brain, lung, and nasopharynx cancers ([34] and references therein). Because of its ability to cross the blood–brain barrier, RLZ is one of the few promising possibilities for glioma therapy [35,36] and brain metastasis in late-stage malignant melanoma [37]. The low systemic bioavailability of RLZ (60% [38,39]) is limited by its poor aqueous solubility (350 mg/L at 37 °C and pH 7 [40]), which is typical for a BCS Class II drug [41]. The potential use of RLZ in psychiatry and oncology stimulates the search for novel forms of this drug, with improved dissolution performance and reduced side effects. During its 20 years of use, many approaches for optimizing the drug delivery of RLZ have been proposed, including salt/cocrystal formation [40,42,43], encapsulation in nanoparticles [44], amorphous form stabilization [45], the use of oral suspensions [46], biodegradable sublingual foils [47], and microemulsions for intranasal delivery [48].

**Scheme 1:**
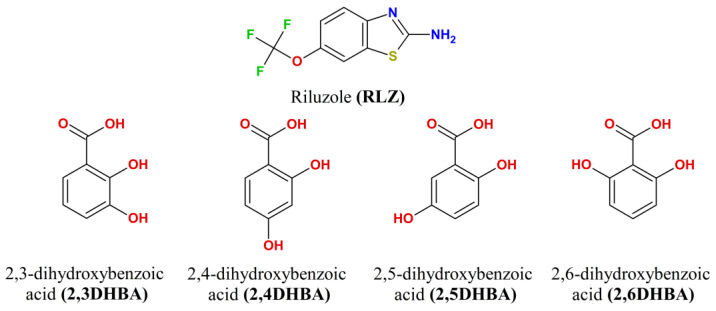
Structural formulas of riluzole and (di)hydroxybenzoic acids considered in this study.

From the crystal engineering point of view, RLZ is known to form multicomponent crystals, with amino and carboxylic acids, aromatic amides, etc. [40,42,43]. Dihydroxybenzoic acids are promising coformers/counterions of RLZ due to their safety and biological activity. 3,4-Dihydroxybenzoic acid (syringic acid) exhibits antibacterial, antifungal, and antiprotozoal activity, 2,5-dihydroxybenzoic acid (gentisic acid) displays antioxidant properties [49], while 2,6-dihydroxybenzoic acid and other derivatives of salicylic acid display anti-inflammatory activity [50] (Scheme 1). 

Based on a survey of multicomponent crystals of RLZ, deposited in the Cambridge Structural Database (CSD) [51] (Supplementary Materials, Section S1), we concluded that in multicomponent crystals with salicylic acid and its hydroxyl derivatives, RLZ exists in the protonated state (as a cation), whereas acids exist in the anionic form. Depending on the position of the OH group in hydroxyl derivatives of salicylic acid, either intra- or intermolecular H-bonds can form. Because RLZ can only generate three H-bonds in the protonated state, the emergence of a new proton donor, with an excess of unshared electron pairs, may cause a dramatic shift in the H-bond network in a multicomponent crystal [52]. In addition, a change in the number of intra- and intermolecular H-bonds can result in a significant change in the lattice energy value [53]. Thus, the position of the second OH group in the salicylic acid derivative is likely to play an essential structure-directing role in riluzole salts. In other words, hydroxyl derivatives of salicylic acid are envisaged as counterions for the design of pharmaceutical salts of riluzole.

This work aims to study the effect of positional isomerism in hydroxyl derivatives of salicylic acid on the H-bond network and supramolecular organization in RLZ salts. Collaborative research, using X-ray diffraction and periodic (solid-state) density functional theory (DFT), was conducted to highlight the critical significance of the mutual arrangement of OH groups in the packing of these multicomponent crystals. 

## 2. Materials and Methods

### 2.1. Experimental

Riluzole of 98% purity was obtained from TCI Chemicals. All the benzoic acid derivatives were purchased from Sigma-Aldrich and were used as received. The purity of materials was checked by differential scanning calorimetry (DSC). The solvents were of analytical or chromatographic grade.

#### 2.1.1. Mechanochemical Experiments

The grinding experiments were performed using a Fritsch planetary micro mill, model Pulverisette 7, in 12 mL agate grinding jars with ten 5 mm agate balls at a rate of 500 rpm for 60 min. In a typical experiment, 50–60 mg of a physical mixture, with a 1:1 molar ratio, was placed into a grinding jar, and 50 μL of solvent (water, methanol, ethanol, acetonitrile, or ethyl acetate) was added with a micropipette. The resulting powder samples were analyzed by powder X-ray diffraction (PXRD), DSC, TGA, and FTIR (see Supplementary Materials, Section S2).

#### 2.1.2. Single Crystal XRD

Single crystal diffraction data were collected using a Bruker D8 Venture diffractometer (Bruker AXS, Karlsruhe, Germany) with graphite-monochromated Mo-Kα radiation (*λ* = 0.71073 Å). It was found that crystals [RLZ+2,4DHBA] degrade below 170 K and thus reflection intensities were collected at 175 K. Absorption corrections, based on measurements of equivalent reflections, were applied [54]. The structures were solved by direct methods and refined by full matrix least-squares on *F*^2^ with anisotropic thermal parameters for all non-hydrogen atoms [55]. In the structure [RLZ+2,6DHBA], the trifluoromethoxy group is disordered over two positions, with occupancy ratio 0.785(3)/0.225(3), and all fluorine atoms were refined with restrained C–F and F···F distances (SADI instruction). All hydrogen atoms were found from difference Fourier synthesis and refined isotropically. Experimental details are listed in Table S2. The crystallographic data were deposited with the Cambridge Crystallographic Data Centre as supplementary publications under the CCDC numbers 2238373, 2238374. This information can be obtained free of charge from the Cambridge Crystallographic Data Centre via www.ccdc.cam.ac.uk/data_request/cif (accessed on 21 February 2023).

### 2.2. Computational Details

#### 2.2.1. Periodic DFT Calculations

B3LYP [56,57] and PBE-D3 [58,59] are the most popular functionals in periodic (solid-state) DFT calculations of molecular crystals [60,61]. B3LYP and PBE-D3 provide a grounded trade-off between the accuracy and the rate of calculations of experimentally observed properties of molecular crystals [62,63,64,65,66,67,68]. Computations were conducted using the CRYSTAL17 package [69]. The 6-31G** basis set was used. The space groups and the unit cell parameters of the crystal obtained from the X-ray diffraction experiment were fixed, and the structural relaxations were limited to the positional parameters of the atoms [70,71]. Tolerance on energy controlling the self-consistent field convergence for geometry optimizations and frequency computations was set to 10^−10^ and 10^−11^ Hartree, respectively. The shrinking factor of the reciprocal space net was set to three. The structures optimized at the PBE-D3/6-31G** level were found to correspond to the potential energy surface’s minimum point. Therefore, PBE-D3 was used in estimating the lattice energy using Equation (3).

#### 2.2.2. Enthalpy of the Intermolecular H-Bonds

The enthalpy Δ*H_HB_* of the intermolecular H-bond in the considered crystals was estimated using the Rozenberg approach [72]:

−Δ*H_HB_* [kJ·mol^−1^] = 0.134·*R*(H∙∙∙O)^−3.05^, (1)

where the *R*(H∙∙∙O) is the H∙∙∙O distance (nm). Equation (1) describes −Δ*H_HB_* varying from 10 to 80 kJ·mol^−1^ [72]. The *R*(H∙∙∙O) values were obtained as a result of geometry optimization.

#### 2.2.3. Estimation of the Crystal Lattice Energy

The energy of intermolecular noncovalent interaction *E_int_* in the considered crystal was evaluated according to ref. [73] as follows:

*E_int_* [kJ·mol^−1^] = 1124·*G_b_* [atomic units] (2)

where *G_b_* is the positively defined local electronic kinetic energy density at the (3,−1) bond critical point, corresponding to the considered intermolecular noncovalent interaction [74]. The methodology of the calculation is presented elsewhere [75]. Periodic wave functions computed at the PBE-D3/6-31G** level were used in Topond14 [76]. Equation (2) overestimates energies for the short (strong) intermolecular H-bonds [77]. Therefore, the use of Equation (1) is preferable for systems with short H-bonds, both in crystals [77] and in solutions [78]. 

On the other hand, Equation (2) enables one to estimate the lattice energy of a two-component organic crystal using the following approach [75]:

*E_latt_* = ∑*_i_*∑*_j<i_*(*E_int,j,i_*), (3)

where indices *j* and *i* denote the atoms belonging to different molecules. Equation (3) is BSSE-free. Details of the *E_latt_* computations in the two-component crystals using Equation (3) are given elsewhere [75]. In the present study, we were interested in the *relative E_latt_* values estimated for riluzole salts. Hence, overestimating the values of *E_int_* given by Equation (2) does not play a significant role.

#### 2.2.4. Molecular Electrostatic Potential 

The molecular electrostatic potential surfaces (MEPs) [79] of the RLZ and selected (di)hydroxybenzoic acids were generated at the B3LYP/def2-tzvp level of theory, with the latest version of the D3 dispersion correction, using Gaussian09 [80]. Calculations were carried out using the optimized molecular geometries. The local maxima and minima sites on the molecular electrostatic potential surfaces were extracted using the Multiwfn software [81].

## 3. Results

In the present work, two crystals of riluzole, namely, RLZ+2,6-dihydroxybenzoic acid [RLZ+2,6DHBA] (1:1) and RLZ+2,4-dihydroxybenzoic acid [RLZ+2,4DHBA] (1:1), were obtained and structurally characterized (see Table S2, Figures S1, S2, S4, S6 and S7). The details of the screening experiments and phase characterization using powder XRD, DSC, thermogravimetric analysis (TGA), and Fourier-transform infrared spectroscopy (FTIR) are given as Supplementary Materials (Sections S2 and S3). The formation of the novel phase was also observed in the RLZ+2,3-dihydroxybenzoic acid system (Figure S3), which was also proved to be a salt, based on its FTIR spectrum (Figure S4) and DSC/TGA profile (Figure S5). However, we were unable to grow single crystals of sufficient size for structural characterization. For comparison, crystalline RLZ+2,5-dihydroxybenzoic acid [RLZ+2,5DHBA] (1:1) and RLZ+salicylic acid [RLZ+2HBA] (1:1), obtained earlier [43], were also considered in the present study.

**Figure 1:**
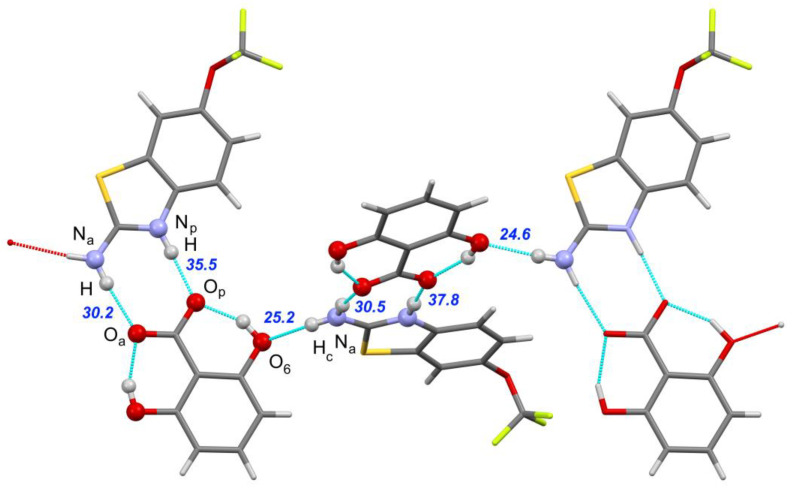
The H-bonded chain in crystalline [RLZ+2,6DHBA] (1:1). The asymmetric unit contains two inequivalent RLZ+2,6DHBA heterodimers. H-bonds are shown as dotted lines. Numbers indicate the enthalpies of intermolecular H-bonds estimated using Equation (1) at the PBE-D3 level. H_c_ denotes the H atom of the amino group of the RLZ molecule in the *cis*-position with respect to the S atom (Scheme 1).

**Figure 2:**
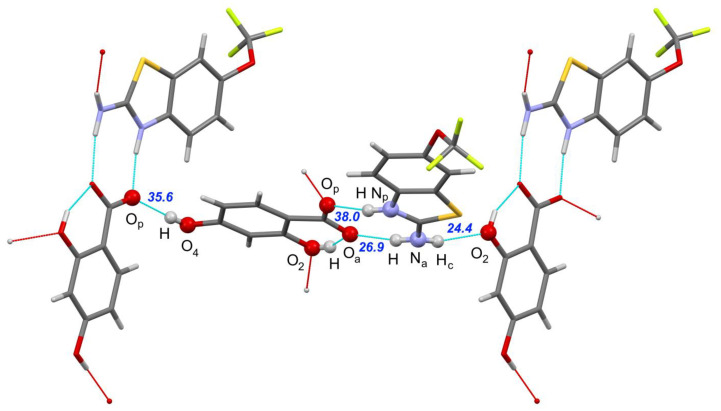
Fragment of the hydrogen bond network in crystalline [RLZ+2,4DHBA] (1:1). H-bonds are shown as dotted lines. Numbers indicate the enthalpies of intermolecular H-bonds estimated using Equation (1) at the PBE-D3 level.

All the crystals under consideration contain a robust amino-N-heterocycle carboxylate supramolecular heterosynthon, of $R_{2}^{2}\left(8\right)$ topology. This synthon is structurally identical to 2-aminopyridinium carboxylate [82], which is known to play a structure-directing role in various multicomponent crystals [83,84,85,86,87,88]. The mutual orientation of molecules in the synthon is determined by the side H-bonds, and varies from coplanar in [RLZ+2,5DHBA] and [RLZ+2,6DHBA] to the twisted one in [RLZ+2HBA] and [RLZ+2,4DHBA]. The addition of a second hydroxyl group in the salicylic acid changes the H-bonding pattern considerably, in a non-systematic manner, see Figure 1 and Figure 2 and Table 1. The second hydrogen of the amino group of RLZ, denoted as N_a_-H_c_, forms in these salts an H-bond with the lone electron pair of the OH group of the neighboring acid molecule, rather than with the oxygen of the CO_2_− group, c.f. Scheme 4 in Ref. [73], Figure 1 and Figure 2. The supramolecular organization in crystalline [RLZ+2,6DHBA] (1:1) differs from that in [RLZ+2,4DHBA] (1:1) and [RLZ+2,5DHBA] (1:1). Because the second OH group is absent in the sixth position, intermolecular charge-assisted hydrogen bonds are formed in the latter crystals (Table 1). This isomerism leads to the appearance of a two-dimensional network of H-bonds, c.f. Figure 1 and Figure 2. The CO_2_− group of the counterion in all RLZ salts, forms four H-bonds. However, the resulting H-bond network is determined by the number of **intramolecular** H-bonds. [RLZ+2,6DHBA] (1:1) is the only crystal with two such H-bonds. As a consequence, 2,6-dihydroxybenzoic acid can form only **three** “unique” intermolecular H-bonds, while other OH derivatives of salicylic acid form **four** intermolecular H-bonds.

As can be seen from the crystal structure data, different positions of the hydroxyl group in the counterion lead to variations in the H-bond topology and mutual orientation of H-bonded units, without significant changes in the interaction energy (Table 1). A structural similarity analysis, performed with the XPac program [89,90], revealed that the salts of RLZ with salicylic and isomeric dihydroxybenzoic acids have no common packing motifs that could be associated with any kind of non-covalent interaction. Such a variety of crystal structures of structurally related compounds, based on a single “basic” supramolecular heterosynthon, highlights the gap between the topology of H-bonds and the actual packing architecture. The differences in the packing of heterodimers observed in the [RLZ+2,4DHBA] salt, and its solvate with methanol [42], are explained by the participation of the solvent molecule in the H-bond network. No similarity was found between [RLZ+2,6DHBA] and the reported [RLZ+2HBA] crystal [43], even taking into account the fact that the number and topology of H-bonds in these crystals are very similar. In the latter structure, the RLZ-acid dimer is twisted from planarity, which greatly affects the packing. The non-planar structure of the dimer allows the keto oxygen atom to participate in a side N–H···O H-bond with the amide group of RLZ. For [RLZ+2,6DHBA], this site is inaccessible for intermolecular contacts due to the strong O–H···O intramolecular bond.

The studied crystals share the same T-shaped mutual orientation of heterodimers in the hydrogen-bonded chain, despite having varying symmetry and packing. In contrast to the two-centered $R_{2}^{2}\left(8\right)$ motif, that facilitates the coplanar orientation of molecules within the heterodimer, the side N–H···O interaction is single-centered, so the heterodimers can rotate around the bond. An indicative parameter of the T-packing is the dihedral angle between the two nearest heterodimers, which varies from 67° in [RLZ+2,6DHBA] to 87° in [RLZ+2HBA]. The difference in packing angles reflects the difference in the mutual positions of donors and acceptors of side H-bonds within the heterodimer and the influence of weak interactions on the packing architecture.

**Figure 3:**
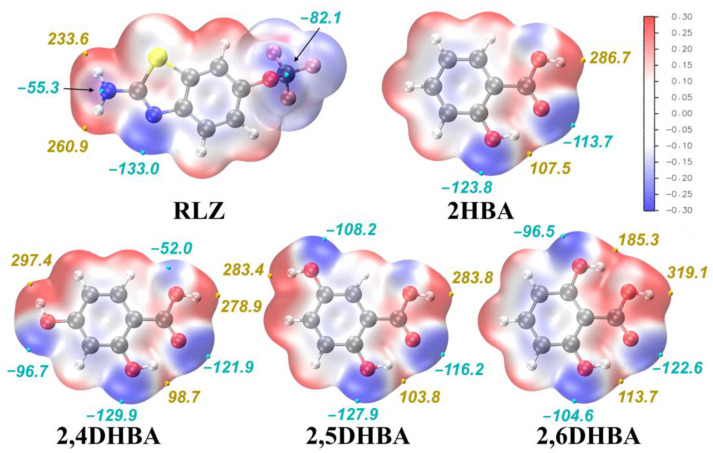
MEP mapped over total electron density, *ρ* = 0.002, isosurface for relaxed molecules of RLZ and the selected counterions. The isosurfaces are color-coded in the range between −0.3 a.u. (blue) and +0.3 a.u. (red). The numbers display the selected electrostatic potential values of minima (cyan points) and maxima (yellow points) of selected electrostatic potentials values, in kJ mol^−1^.

The variety of H-bonds formed in the salts under study can be rationalized based on the comparison of the minima on the MEP surface of RLZ and its counterions (Figure 3). The relative strength of the acceptor sites at the hydroxyl oxygen atom in the *ortho*-position (O2), is larger than that of hydroxyl oxygens in other positions (O4 and O5), which makes it a preferred target for H-bonding with the N_a_–H_c_ donor group remaining after the main heterosynthon is formed.

According to the results of the MEP analysis, the molecules have several donor and acceptor sites, that may be rated by strength in accordance with their electrostatic potential values. The isolated RLZ molecule has three acceptors and two H-bond donors, with the heterocyclic nitrogen being considerably stronger than the ether oxygen and the amine nitrogen. 2HBA has one strong donor and two acceptor sites at the carbonyl oxygen and hydroxyl oxygen atoms, while dihydroxybenzoic acids have three strong acceptor sites. Due to the formation of the second intramolecular hydrogen bond, the number of available donors and acceptors in the 2,6DHBA molecule is lower by one than in 2,4DHBA and 2,5DHBA. The competition between acceptor sites with close MEP values at the carbonyl oxygen atom of the carboxylic group and the oxygen atom of the side hydroxyl groups, is observed in the (di)hydroxybenzoic acids. In 2,4DHBA and 2,5DHBA, the hydroxyl oxygen in the *ortho*-position is stronger than the oxygen atoms of the free hydroxyl groups.

Due to steric factors, the preferred interaction in the crystal is the carboxyl–amino-N-heterocycle heterosynthon [82,91] with the two strongest donor/acceptor sites in the RLZ molecule, e.g., N_a_–H and N_p_–H (for atom labeling see Figure 1 and Figure 2). Compared to (di)hydroxybenzoic acids, RLZ has only one good acceptor site (the N atom of the thiazole ring), which becomes a donor after protonation. After the main amino-N-heterocycle carboxylate heterosynthon is formed, RLZ can only participate in the H-bonding, since its remaining acceptors (the lone pairs at the –OCF_3_ oxygen and the –NH_2_ nitrogen) have lower absolute electrostatic potential values than the acceptor sites of the hydroxybenzoic acids. The formation of H-bonds occurs in accordance with Etter’s third rule, as the strongest donors bind to the strongest available acceptors [92]. The remaining strong donor in the RLZ molecule (N_a_–H_c_) is then attached to the stronger of two competing H-bond acceptors: the O2 atom in the salts with 2,4DHBA and 2,5DHBA, and the O6 atom in the salt with 2,6DHBA. 

The enthalpies, Δ*H_HB_*, of the intermolecular H-bonds in the considered crystals, evaluated using Equation (1), are given in Table 1. As follows from the presented data, the total enthalpy of the amino-N-heterocycle carboxylate supramolecular heterosynthon (65–70 kJ·mol^−1^) does not change significantly depending on the number of OH groups in the substituted benzoic acid or the number of intramolecular hydrogen bonds. The obtained values of the total enthalpy are in good agreement with the available literature data [42,52,93]. On the other hand, the [RLZ+2,4DHBA] (1:1) and [RLZ+2,5DHBA] (1:1) salts feature an additional relatively short O–H···O^−^ bond, with an enthalpy above 30 kJ·mol^−1^ (Table 1). As a result, the total enthalpy of intermolecular H-bonds in [RLZ+2,6DHBA] (92 kJ·mol^−1^) is lower compared to that of [RLZ+2,4DHBA] (125 kJ·mol^−1^) and [RLZ+2,5DHBA] (130 kJ·mol^−1^), because of the lower number of intermolecular H-bonds. It follows from Table 1, that both approaches reproduce the experimental values of distances between heavy atoms involved in intermolecular H-bonds quite well.

The total energy of H-bonds is about 45% of the crystal lattice energy of pharmaceutical multicomponent crystals [63,65,94]. This fact suggests that the energy of the [RLZ+2,6DHBA] crystal lattice is significantly lower than the corresponding values for [RLZ+2,4DHBA] and [RLZ+2,5DHBA] (Table 2). The point is, that the lattice energy of [RLZ+2,6DHBA] is lower than that of [RLZ+2HBA].

## 4. Discussion

The formation of salts in the RLZ-2,6DHBA and RLZ-2,4DHBA systems supports our hypothesis on the influence of the pK_a_ of components on governing the ionization state. As seen from Table S1, the ΔpK_a_ values for RLZ-2,6DHBA and RLZ-2,4DHBA pairs are positive, which agrees with the trend observed for the known RLZ crystals. In brief, hydroxybenzoic acids with –OH groups in positions other than 2 and 6 have pK_a_ values larger than protonated RLZ, and form cocrystals, whereas *ortho*-hydroxybenzoic acids result in positive ΔpK_a_ with RLZ, and form salts.

Although the solubility of a single-component organic compound can be estimated using the Yalkowsky equation [95], or other QSAR models [96,97], with a fair degree of precision, forecasting the solubility of pharmaceutical salts containing different types of counterions is not a trivial task. In fact, only a handful of studies have looked at this issue in depth, examining the effect of structural and physicochemical features of organic salts on their solubility in a systematic manner [98,99,100,101]. The generally accepted view on this topic, implies that the change in the free energy of salt dissolution can be represented as the sum of contributions from the values of the free energy of hydration of the cation and anion, as well as the free energy of the crystal [101,102]:

Δ*_sol_G*(*salt*) = Δ*_hyd_G*(*cation*) + Δ*_hyd_G*(*anion*) − Δ*_latt_G*(*salt*), (4)

In the case of salts of riluzole with hydroxyl derivatives of salicylic acid, the first term on the right side of Equation (4) is a constant, and the second term can also be considered a constant in the first approximation. Thus, the differences in the solubility of the considered salts are mainly due to the third contribution on the right side of Equation (4). From the point of view of the “enthalpy-determined drug solubility processes” [103], Δ*_latt_G*(*salt*) can be replaced by Δ*_latt_H*(*salt*). The latter value is proportional to the crystal lattice energy of the salt. Given that [RLZ+2,6DHBA] has the lowest crystal lattice energy among all the salts under consideration, it might be reasonable to assume that [RLZ+2,6DHBA] is likely to have a better solubility compared to other salts. This conclusion is consistent with the literature data. For example, the solubility of the ethionamide salt with 2,6DHBA, was found to be higher than that with 2,3-dihydroxybenzoic and 2,4-dinitrobenzoic acids [104]. To summarize, 2,6DHBA appears to be a promising compound to consider during cocrystal or salt screening trials, due to its ability to modify the crystalline environment of the target drug without compromising total lattice energy gain and, thus, solubility.

## 5. Conclusions

In this work, specific features of hydrogen bond topology, induced by the introduction of a second OH group into the counterion molecule, were revealed through a detailed structural analysis of multicomponent crystals of riluzole with 2-hydroxybenzoic acid and 2,4-, 2,5-, and 2,6-dihydroxybenzoic acids. In the solid forms containing 2-hydroxybenzoic acid and 2,6-dihydroxybenzoic acid, the hydrogen bonding network is sustained solely by the N–H groups of riluzole, resulting in the formation of 1D chains. The competition between the acceptor centers of the counterion causes a change in the topology of hydrogen bonds and differences in the packing arrangements of salts with 2-hydroxybenzoic and 2,6-dihydroxybenzoic acids. The presence of an additional H-bond donor at position 4 or 5, leads to the formation of charge-assisted H-bonds and the combination of isolated hydrogen-bonded chains into 2D sheets. 

According to periodic DFT calculations, the number and position of the OH-groups in the counterion molecule appear to have little effect on the enthalpy of the primary supramolecular synthon (65–70 kJ·mol^−1^). A novel intermolecular charge-assisted H-bond in the salts containing 2,4- and 2,5-dihydroxybenzoic acids, stabilizes the corresponding crystal structure and increases the lattice energy by about 30 kJ·mol^−1^. When compared to riluzole salts with 2,4- and 2,5-dihydroxybenzoic acids, the salt with 2,6-dihydroxybenzoic acid lacks an intermolecular charged-assisted O–H···O^−^ H-bond, resulting in a lower lattice energy and expected higher solubility. In addition, 2,6-dihydroxybenzoic acid is preferred over salicylic acid, because the lattice energy of riluzole salt with 2,6-dihydroxybenzoic acid is lower than that of riluzole salt with 2-hydroxybenzoic acid. We do hope that this prediction encourages experimentalists to conduct further investigations of riluzole multicomponent crystals with dihydroxybenzoic acids, especially 2,6-dihydroxybenzoic acid.

## Figures and Tables

**Table 1:** The difference between the calculated (B3LYP/6-31G** and PBE-D3/6-31G**) and experimental values of the distances between heavy atoms Δ*D* in the considered crystals. The enthalpies Δ*H_HB_* (kJ·mol^−1^) of these H-bonds, evaluated using the Rozenberg approach, see Equation (1). The PBE-D3 values are given in italics.

	[RLZ+2HBA]		[RLZ+2,6DHBA] ^(b)^		[RLZ+2,5DHBA]		[RLZ+2,4DHBA]	
Fragment ^(a)^	Δ*D*, Å	−Δ*H_HB_*	Δ*D*, Å	−Δ*H_HB_*	Δ*D*, Å	−Δ*H_HB_*	Δ*D*, Å	−Δ*H_HB_*
N_p_–H∙∙∙O_p_	0.046 *0.003*	32.1 *36.3*	−0.026 −*0.024*	35.8 *36.6*	0.001 −*0.038*	34.4 *38.7*	−0.001 −*0.016*	35.4 *38.0*
N_a_–H∙∙∙O_a_	0.009 −*0.017*	29.3 *30.3*	−0.008 −*0.028*	28.5 *30.4*	0.004 −*0.005*	30.1 *31.1*	0.014 −*0.002*	25.6 *26.9*
N_a_–H_c_∙∙∙O’_p_	0.002 −*0.017*	27.4 *28.8*	-	-	-	-	-	-
N_a_–H_c_∙∙∙O’_6_	-	-	0.082 *0.030*	22.5 *24.6*	-	-	-	-
N_a_–H_c_∙∙∙O’_2_	-	-	-	-	−0.022 −*0.051*	26.9 *28.8*	−0.019 −*0.042*	22.7 *24.4*
O’_5_–H∙∙∙O_p_	-	-	-	-	−0.021 −*0.042*	29.7 *31.9*	-	-
O’_4_–H∙∙∙O_p_	-	-	-	-	-	-	−0.019 −*0.051*	32.6 *35.6*

^(a)^ See Figure 1 and Figure 2 for atom numbering; H_c_ denotes the H atom of the amino group of the RLZ molecule in the *cis*-position with respect to the S atom (Scheme 1). ^(b)^ The values for [RLZ+2,6DHBA] are averaged over two heterodimers in the asymmetric unit.

**Table 2:** Total energy of intermolecular H-bonds in the crystals of RLZ salts obtained using Equation (2), and lattice energy estimated using Equation (3). Geometric parameters were calculated at the PBE-D3 level. The units are kJ·mol^−1^.

Crystal	[RLZ+2HBA]	[RLZ+2,4DHBA]	[RLZ+2,5DHBA]	[RLZ+2,6DHBA]
Σ*E_HB_*	95.4	124.9	130.7	91.6
*E_latt_*	229.0	281.9	266.0	209.4

## Data Availability

Samples of salts obtained in this study, PXRD, DSC, and FTIR data and I/O files are available from the respective author upon reasonable request. Crystal structures of [RLZ+2,4DHBA] and [RLZ+2,6DHBA] have been deposited with the Cambridge Crystallographic Data Centre, under the CCDC numbers 2238373-2238374.

## Supplementary Materials

The following supporting information can be downloaded at: https://www.mdpi.com/article/10.3390/pharmaceutics15030878/s1, Section S1: Analysis of the literature data on crystal structures of riluzole multicomponent crystals with benzoic acid derivatives; Table S1: ΔpKa and hydrogen bond parameters in known crystals of riluzole with benzoic acid derivatives; Section S2: Details of PXRD, DSC, TGA, and FTIR experiments; Section S3: Phase identification and crystal structure analysis; Figures S1–S3: PXRD patterns of RLZ mixtures with dihydroxybenzoic acids after liquid-assisted grinding; Table S2: Crystallographic data for [RLZ+2,4DHBA] and [RLZ+2,6DHBA]; Figure S4: FTIR spectra of RLZ and its salts; Figures S5–S7: DSC and TGA traces of RLZ salts. References [105,106] are cited in the supplementary materials.
