# Supplementary material for: Supramolecular Organization in Salts of Riluzole with Dihydroxybenzoic Acids—The Key Role of the Mutual Arrangement of OH Groups
Source: Pharmaceutics. 2023 Mar 8;15(3):878. doi: 10.3390/pharmaceutics15030878 (PMC10051219; doi:10.3390/pharmaceutics15030878)
Supplement: Supplementary file 1 [file pharmaceutics-15-00878-s001.zip › pharmaceutics-2232366-supplementary.pdf]

Supramolecular Organization in Salts of Riluzole with Dihydroxybenzoic Acids—The Key Role of the Mutual Arrangement of OH Groups

Alexander P. Voronin [1], Artem O. Surov [1], Andrei V. Churakov [2] and Mikhail V. Vener [2,*]

[1] G.A. Krestov Institute of Solution Chemistry RAS, 153045 Ivanovo, Russia

[2] Kurnakov Institute of General and Inorganic Chemistry, Russian Academy of Sciences, Leninskii prosp. 31, 119991 Moscow, Russia

* Correspondence: vener@igic.ras.ru

Section S1. pKa vs proton transfer: literature analysis

A proton transfer from the carboxylic group of the coformer molecule to the nitrogen atom of the thiazole group of riluzole (RLZ) is possible depending on the pK_a difference between constituents of a multicomponent crystal. We have considered a row of five multicomponent crystals of RLZ with –OH and –OCH_3-substituted benzoic acids taken from Cambridge Structural Database (CSD), namely, salicylic acid, 2,4-dihydroxybenzoic acid, 2,5-dihydroxybenzoic acid, vanillic acid, 3,4,5-trimethoxybenzoic acid. The H-bonding parameters and ΔpK_a values for these compounds are presented in Table S1. It is easy to notice that in the systems with $\Delta pK_a < 0$ there is no proton transfer between components, and the forming multicomponent crystal is a cocrystal. At $\Delta pK_a > 0$ the molecules in crystal become ionized, and the salt is formed. The *ortho*-hydroxybenzoic acids are known to have lower pK_a values compared to other isomers due to intramolecular H-bond in their structure. In our case, all three coformers which form salts with riluzole are *ortho*-hydroxybenzoic acids.

Based on the performed CSD survey of multicomponent crystals of RLZ, one can propose an empirical rule, according to which the substituted benzoic acids with hydroxyl fragment in the *ortho* position form salts with RLZ, while isomeric *meta* and *para* isomers either form neutral cocrystals or do not cocrystallize with RLZ at all.

The calculated ΔpK_a value between pyridine group of RLZ and the carboxylic group of the coformer molecules implies the proton transfer between the component molecules in crystal [1]. To prove this hypothesis, we have conducted cocrystallization experiments with three hydroxyl-derivatives of salicylic acid, namely, 2,3-dihydroxybenzoic, 2,4-dihydroxybenzoic, and 2,6-dihydroxybenzoic acids.

Table S1. A summary of hydrogen bonds in the reported multicomponent crystals of RLZ with benzoic acid derivatives. The atom labeling is consistent to corresponding entries in the CSD.

System	ΔpK_a	H-bonded fragment	D···A distance, Å	D-H···A angle, °
Literature data:				
[RLZ + 2,4DHBA methanol solvate] (1:1:1)<br>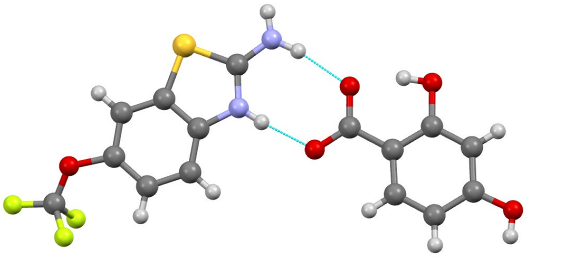	0.69	N2-H2N···O3<br>N1-H1N···O2<br>O6-H3O···O4<br>N2-H3N···O6<br>O4-H1O···O3<br>O5-H2O···O2	2.783(4)<br>2.665(3)<br>2.819(4)<br>2.740(5)<br>2.510(4)<br>2.668(4)	169(4)<br>174(4)<br>172(5)<br>168(4)<br>161(4)<br>177(4)
[RLZ + 2,5DHBA] (1:1)<br>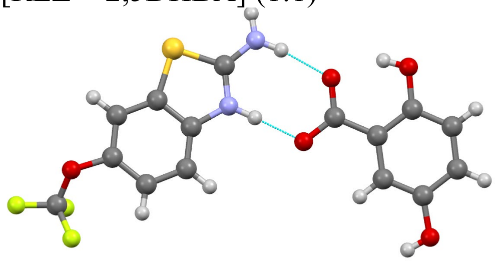	0.83	N1-H2···O4<br>N1-H1···O3<br>N2-H6···O2<br>O2-H8···O5<br>O4-H7···O3	2.802(6)<br>2.731(6)<br>2.683(6)<br>2.690(5)<br>2.572(6)	170(5)<br>169(5)<br>172(5)<br>159(5)<br>146(5)
[RLZ + 2HBA] (1:1)<br>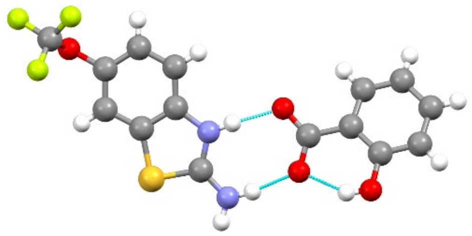	0.83	N1-H2···O2<br>N1-H1···O3<br>N2-H3···O2<br>O3-H7···O4	2.775(5)<br>2.748(5)<br>2.670(5)<br>2.582(5)	175(3)<br>165(4)<br>170(3)<br>135(1)
[RLZ + vanillic acid] (1:1)<br>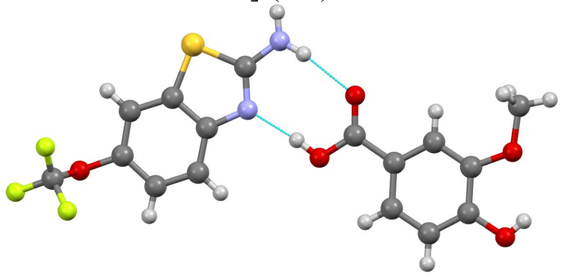	-0.71	O2-H10···N1<br>N2-H1N···O3<br>N2-H2N···O3<br>O4-H2O···F2	2.671(3)<br>2.908(4)<br>2.988(3)<br>3.126(4)	172(4)<br>170(3)<br>152(3)<br>142(4)
[RLZ + 3,4,5-trimethoxybenzoic acid] (1:1)<br>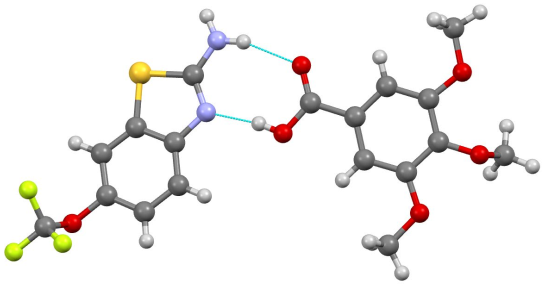	-0.12	N1-H1A···O2<br>O3-H3···N2<br>N1-H1B···O2	2.918(3)<br>2.604(2)<br>2.878(3)	162(2)<br>169(2)<br>135(2)

System	ΔpK[a]	H-bonded fragment	D···A distance, Å	D-H···A angle, °
Structures from this work:				
[RLZ+2,6DHBA] (1:1)	2.50	N11-H10···O31	2.6322(19)	178(2)
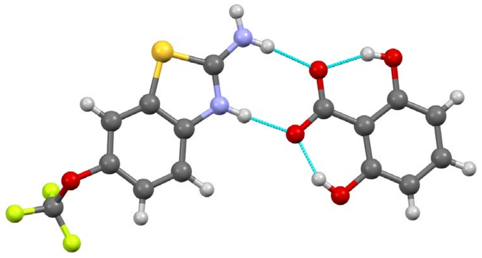		N12-H11···O32	2.766(2)	170(2)
		N21-H20···O41	2.688(2)	177(2)
		N22-H21···O42	2.766(2)	174(2)
		O33-H31···O31	2.4995(18)	154(2)
		O34-H32···O32	2.5503(19)	152(2)
		O43-H41···O41	2.5024(18)	155(2)
		O44-H42···O42	2.556(2)	154(3)
		N22-H22···O33	2.792(2)	166(2)
		N12-H12···O43	2.854(2)	165(2)
[RLZ+2,4DHBA] (1:1)	0.69	N1-H11···O12	2.799(2)	170(2)
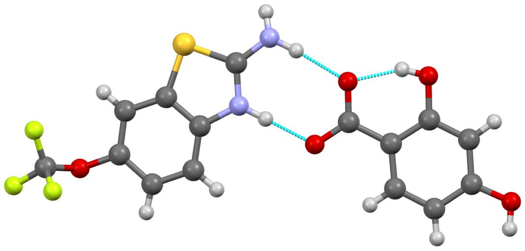		N1-H12···O13	2.854(2)	163(2)
		N2-H2···O11	2.659(2)	170(2)
		O13-H13···O12	2.515(2)	153(3)
		O15-H15···O11	2.6596(18)	171(3)

Section S2. Experimental

Powder X-ray Diffraction (XRD)

The laboratory powder XRD data of the bulk materials were recorded under ambient conditions on a D2 Phaser (Bragg-Brentano) diffractometer (Bruker AXS, Germany) with a copper X-ray source ($\lambda_{CuK\alpha1}$ = 1.5406 Å) and a LYNXEYE XE-T high-resolution position-sensitive detector. The samples were placed into the plate sample holders and rotated at a rate of 15 rpm during the data acquisition.

Differential scanning calorimetry (DSC)

The thermal analysis was carried out using a differential scanning calorimeter with a refrigerated cooling system (*Perkin Elmer* DSC 4000, USA). The sample was heated in a sealed aluminum sample holder at a rate of 10 °C·min^{-1} in a nitrogen atmosphere. The unit was calibrated by indium and zinc standards. The accuracy of the weighing procedure was ± 0.01 mg.

Thermogravimetric analysis (TGA)

The TGA was performed on a TG 209 F1 Iris thermomicrobalance (Netzsch, Germany). Approximately 10 mg of the sample were added to a platinum crucible. The samples were heated at a constant heating rate of 10 °C·min^{-1} and purged throughout the experiment with a dry argon stream at 30 ml·min^{-1}.

Fourier transform infrared spectroscopy (FTIR)

The Fourier-transform infrared spectra of pure RLZ and its salts with dicarboxylic acids were recorded on a Bruker Vertex V80 FTIR spectrometer. All the samples were pressed into KBr pellets and recorded at frequencies from 4000 to 350 cm^{-1}.

Section S3. Identification and crystal structure analysis

Screening for novel crystalline phases of RLZ with dihydroxybenzoic acids was performed using liquid-assisted grinding in the presence of various solvents in the 1:1 ratio using the *Pulverisette 7* planetary micromill. For the RLZ+ 2,6DHBA system, the formation of the same crystalline phase characterized by diffraction peaks at 2θ = 7.6, 9.7, 16.8 and 19.5° was observed in all experiments (Fig. S1). The absence of reflections corresponding to unreacted excess of any of pure components confirms that the stoichiometry of the cocrystals is (1:1).

Since a methanol solvate is known to form in the RLZ+ 2,4DHBA system, the set of solvents was changed to exclude alcohols and other protonic solvents. The diffraction quality of samples obtained in ethyl acetate, acetonitrile and chloroform was found to be variable, with [RLZ+2,4DHBA] obtained from EtOAc displaying the highest crystallinity.

By grinding the RLZ-2,3DHBA mixture in the presence of methanol we were able to obtain a novel crystalline phase different from the starting solids with diffraction peaks at 2θ = 7.1, 7.6, 9.7, 13.2, 14.3, 15.7, 15.9, 22.3, and 28.9°. Recrystallization of the powder in the same solvent led to appearance of long needle-shaped crystals not suitable for diffraction experiment.

At the next step, the single crystals of [RLZ+2,6DHBA] (1:1) and [RLZ+2,4DHBA] (1:1) were obtained using solvent evaporation technique from methanol and ethyl acetate, respectively. Since the size of crystals was too small to perform SC-XRD studies, cyclic heating of the saturated solution with crystals to 45 °C followed by slow cooling to room temperature was performed several times to improve crystal quality and size to diffraction grade.

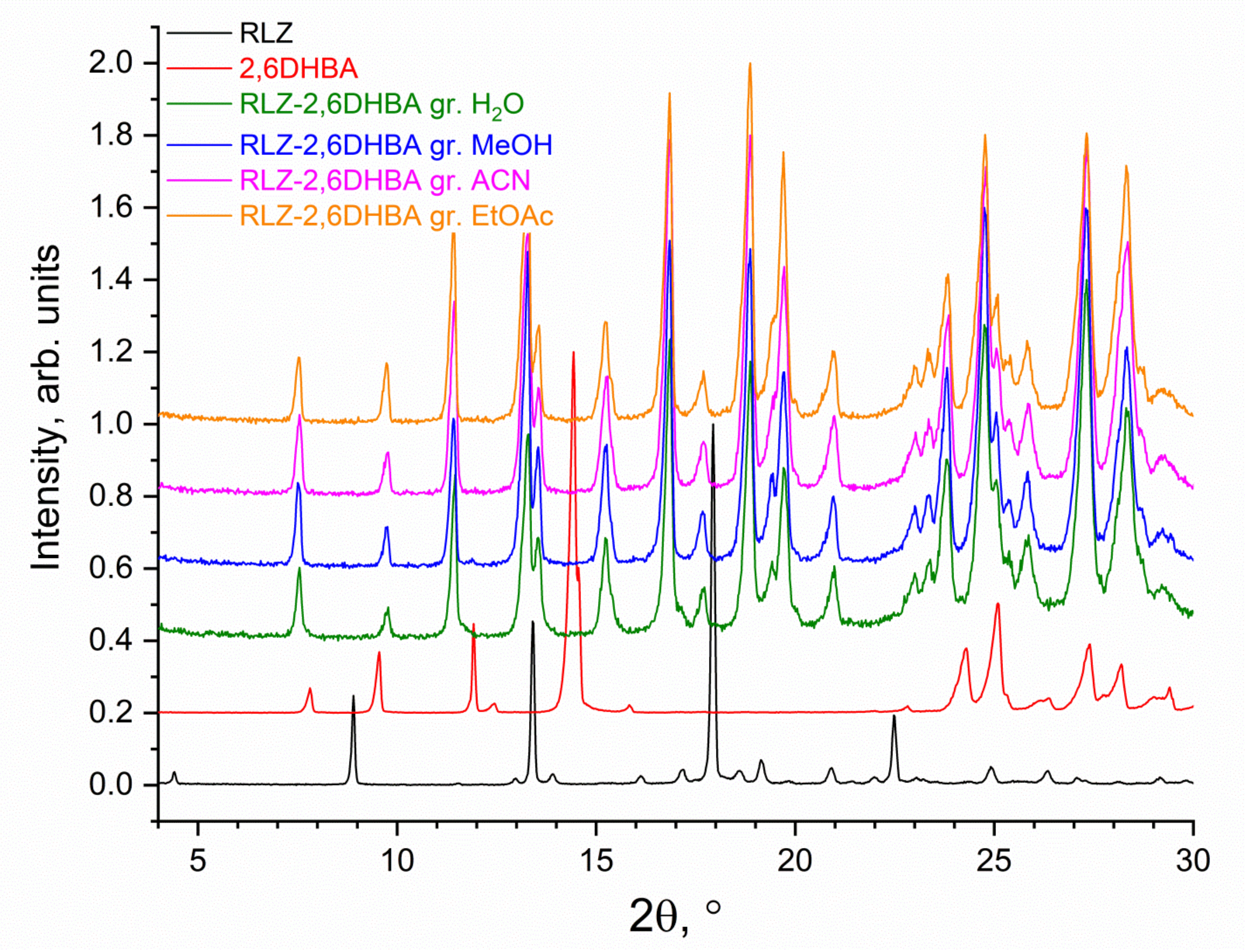

Figure S1. Powder XRD patterns of the [RLZ+2,6DHBA] (1:1) salt obtained using liquid-assisted grinding in the presence of various solvents (water, methanol, ethanol, ethyl acetate) compared to initial RLZ (black line) and 2,6DHBA (red line).

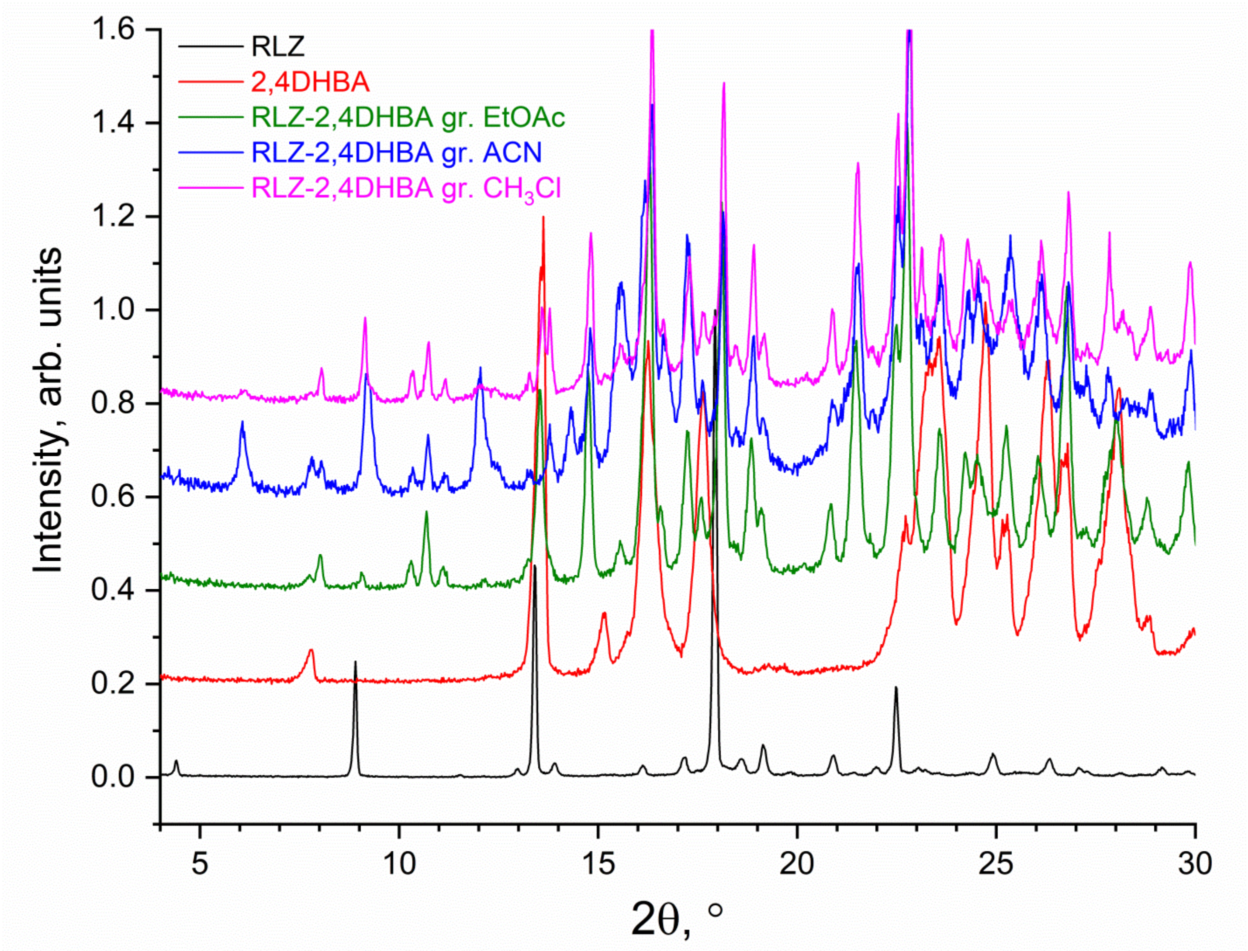

Figure S2. Powder XRD patterns of the [RLZ+2,4DHBA] (1:1) salt obtained using liquid-assisted grinding in the presence of various solvents (ethyl acetate, acetonitrile, chloroform) compared to initial RLZ (black line) and 2,4DHBA (red line).

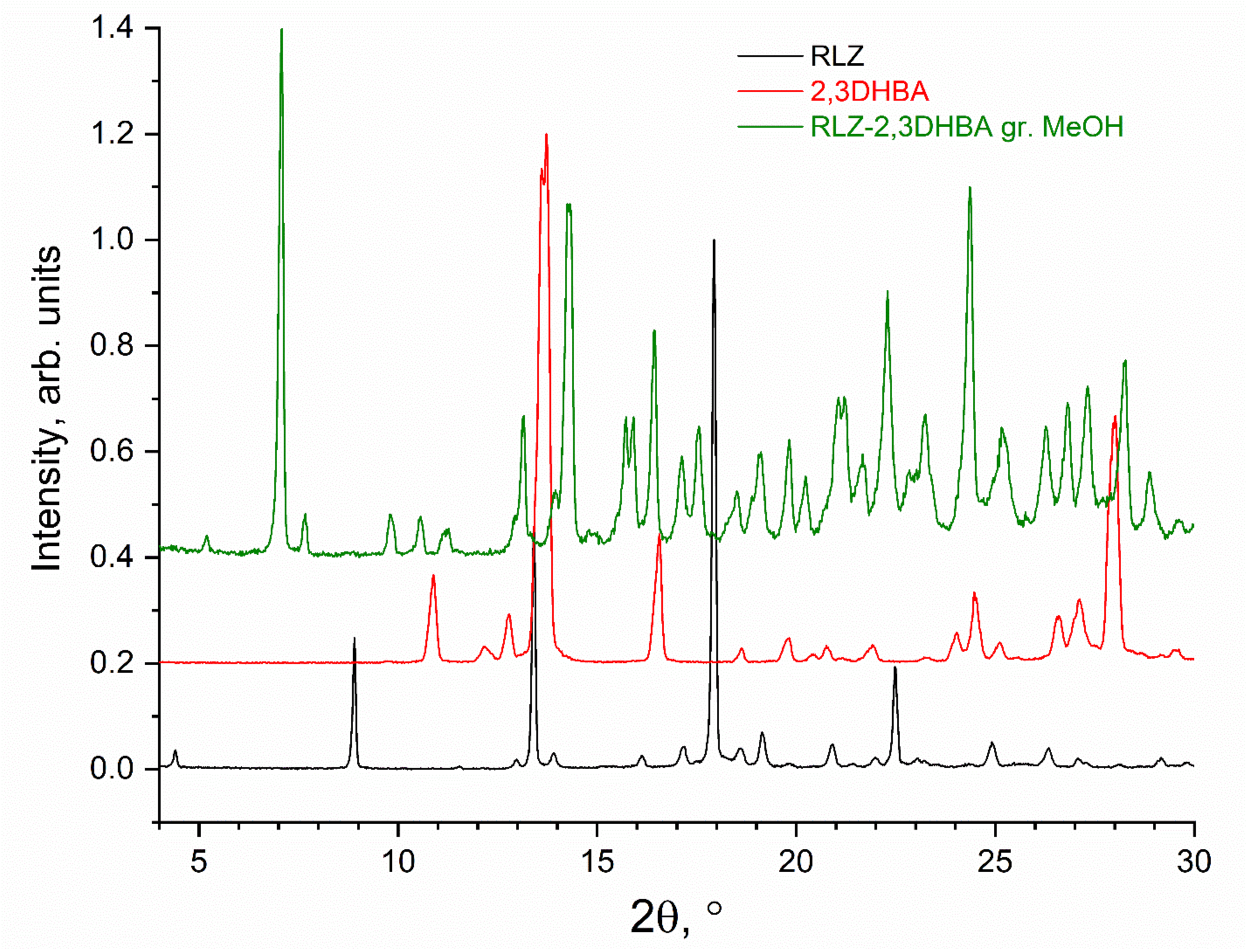

Figure S3. Powder XRD patterns of the [RLZ+2,3DHBA] (1:1) salt obtained using liquid-assisted grinding in the presence of methanol compared to initial RLZ (black line) and 2,3DHBA (red line).

The crystal structures of [RLZ+2,4DHBA] (1:1) and [RLZ+2,6DHBA] (1:1) were solved at the Centre of IGIC RAS (Moscow). The [RLZ+2,4DHBA] (1:1) single crystal was found to degrade at temperatures below 170 K, so the reflections were collected at lowest possible temperature (175 K). Crystallographic data for both salts are presented in Table S2.

The asymmetric unit of the [RLZ+2,4DHBA] (1:1) salt contains one protonated riluzole molecule and one 2,4-dihydroxybenzoate molecule forming a robust heterosynthon. The –OCF_3 group or RLZ in the crystal is disordered over two positions with occupancy values 0.78 / 0.22. In contrast to [RLZ+2,4DHBA] (1:1), the asymmetric unit in the [RLZ+2,6DHBA] (1:1) salt contains two symmetry-inequivalent RLZ molecules without disorder which differ by the rotation angle of the –OCF_3 group with respect to the planar fragment of the molecule and two 2,6DHBA molecules with identical conformation. As expected from the CSD survey (Section S1), a proton transfer occurs in both crystals.

Table S2. Crystallographic data, details of the SC-XRD experiment and structure refinement for riluzole salts with dihydroxybenzoic acids obtained in this study

	[RLZ+2,4DHBA] (1:1)	[RLZ+2,6DHBA] (1:1)
CCDC No.	2238373	2238374
Crystal data		
Chemical formula	$C_8H_6F_3N_2OS·C_7H_5O_4$	$C_8H_6F_3N_2OS·C_7H_5O_4$
M_r	388.32	388.32
Crystal system, space group	Monoclinic, $P2_1/n$	Triclinic, $P\bar{1}$
Temperature (K)	174	100
a (Å)	11.5271 (4)	7.9605 (4)
b (Å)	11.0671 (4)	14.2318 (7)
c (Å)	12.7225 (4)	15.5222 (8)
α (°)	90	80.5695 (18)
β (°)	96.0054 (11)	75.7788 (17)
γ (°)	90	75.1738 (16)
V (Å^3)	1614.12 (10)	1638.30 (14)
Z	4	4
Radiation type	Mo $K\alpha$	Mo $K\alpha$
μ (mm^{-1})	0.27	0.26
Crystal size (mm)	0.25 × 0.20 × 0.20	0.22 × 0.18 × 0.03
Data collection		
Diffractometer	Bruker D8 Venture	Bruker D8 Venture
Absorption correction	Multi-scan<br>*SADABS*(Bruker, 2016)	Multi-scan<br>*SADABS* (Bruker, 2016)
No. of measured reflections	20677	16963
Independent reflections	3526	5789
Reflections with [$I > 2\sigma(I)$]	2995	5206
R_{int}	0.032	0.020
$(\sin \theta/\lambda)_{max}$ (Å^{-1})	0.639	0.596
Refinement		
$R[F^2 > 2\sigma(F^2)]$, $wR(F^2)$, S	0.041, 0.103, 1.04	0.034, 0.081, 1.05
No. of reflections	3526	5789
No. of parameters	310	558
No. of restraints	30	1
H-atom treatment	All H-atom parameters refined	All H-atom parameters refined
$\Delta\rangle_{max}$, $\Delta\rangle_{min}$ (e Å^{-3})	0.55, -0.69	0.98, -0.25

The analysis of IR spectroscopic data presented in Fig. S4 supports the proton transfer in all obtained salt forms of riluzole, including [RLZ+2,3DHBA] (1:1). Parent riluzole showed characteristic N-H stretching bands at 3371 and 3278 cm^{-1} in the FTIR spectrum (Fig. S4a) which can be assigned to the primary amino group. [2] The absence of strong carboxylic O-H stretching bands at 3600-3400 cm^{-1} typical for RLZ cocrystals with carboxylic acids [2,3] indicates the absence of the molecular forms of carboxylic acid in the crystal, which agrees with SC-XRD data. The carboxylate C=O stretching peak around 1400 cm^{-1} is located at the same wavelength for all three multicomponent crystals, suggesting an identical ionization state of this fragment. In addition, the N-H stretching and bending peaks are shifted in the FTIR spectra of novel solid forms by around 50 cm^{-1} compared to pure RLZ.

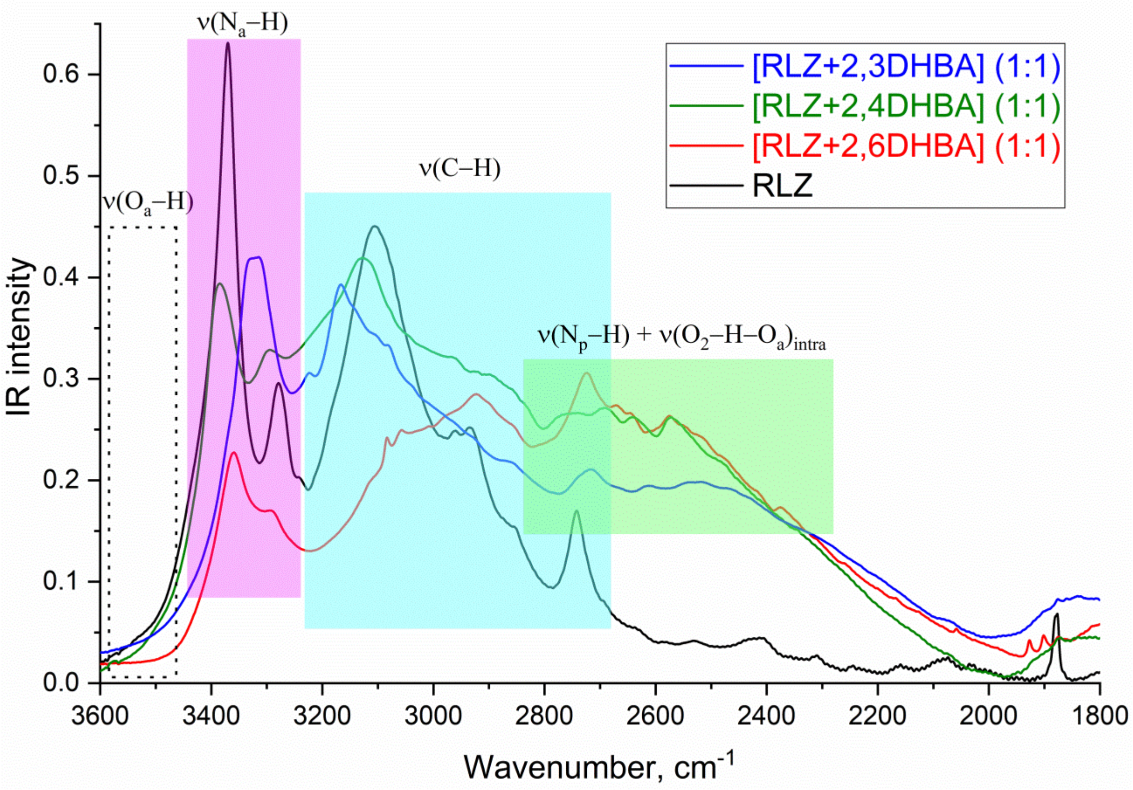

(a)

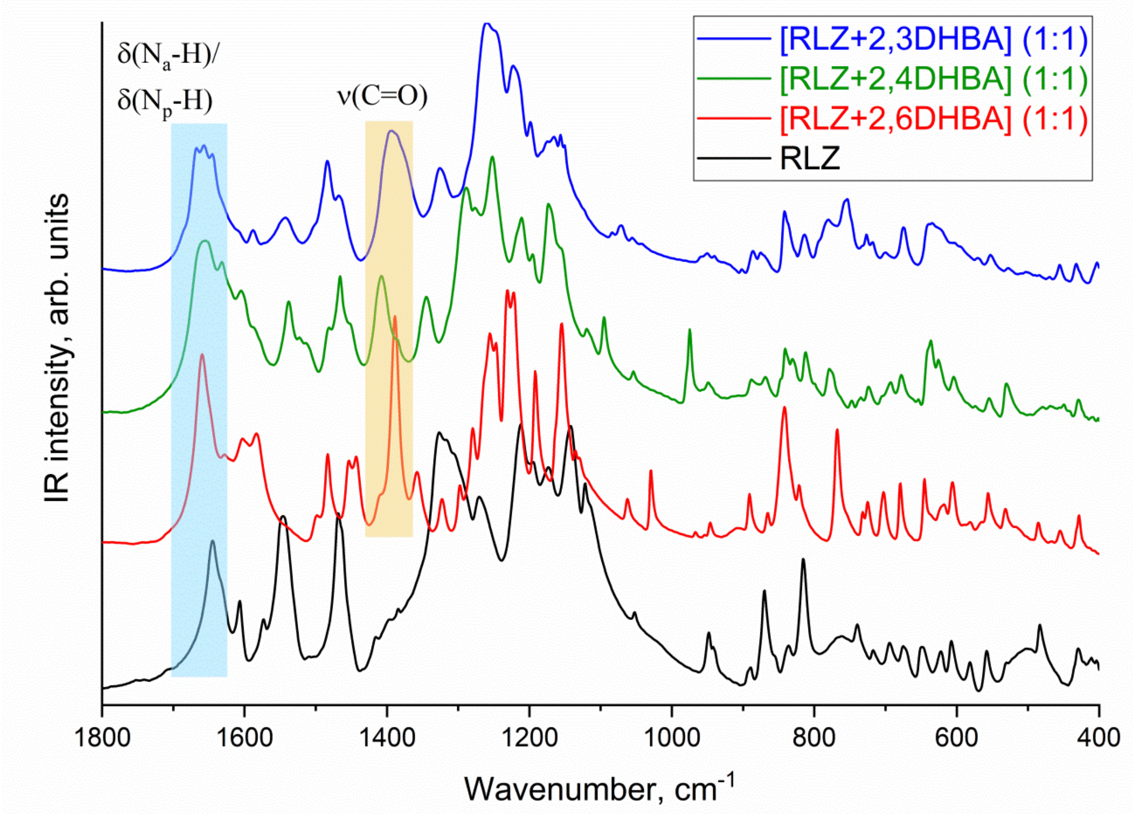

(b)

Figure S4. Overlay of the FTIR absorbance spectra of RLZ and its novel solid forms in KBr pellets: (a) in the 3600-1800 cm^{-1} area; (b) in the 1800-400 cm^{-1} area. The atom labeling is shown in Figures 1 and 2. Note the absence of strong $\nu(O_a$-H) bands typical for IR spectra of RLZ cocrystals with carboxylic acids. [2]

The melting of the RLZ salts is accompanied with decomposition, as shown in Fig. S5-S7, which does not allow one to estimate precisely the fusion enthalpy. The [RLZ+2,3DHBA] (1:1) and [RLZ+2,4DHBA] (1:1) salt undergo phase transition at 145°C and 135°C, respectively, while [RLZ+2,6DHBA] (1:1) remains solid for up to 190°C. Right after melting, all samples experience significant (> 90%) weight loss that may be associated with gas formation or evaporation. All three salts have higher melting temperature compared to parent riluzole (118°C), which is consistent with reported melting points of other salt forms of RLZ [3]. Since no weight loss or other thermal events were recorded prior melting, the solid samples can be considered unsolvated and stable.

Since there are no reports on thermal decomposition of parent RLZ and dihydroxybenzoic acids during melting, [3,4] we believe that a chemical reaction occurs between these compounds in the molten state. Thus, the unexpected superior thermal stability of the salt with 2,6DHBA compared to other studied crystals is not associated with crystal lattice energy (Table S3) and possibly reflects the activation energy of said reaction.

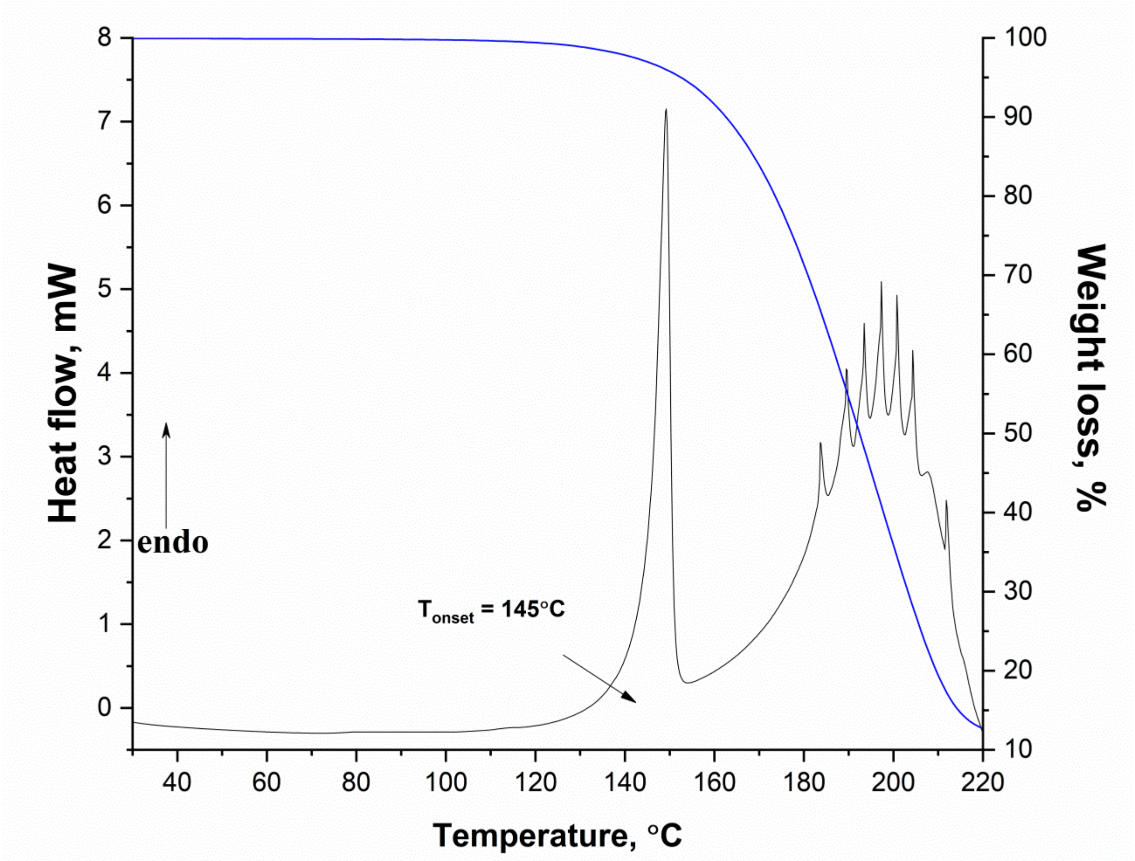

Figure S5. DSC (black line) and TGA thermograms (blue line) of [RLZ+2,3DHBA] recorded at 10 °C min^{-1}.

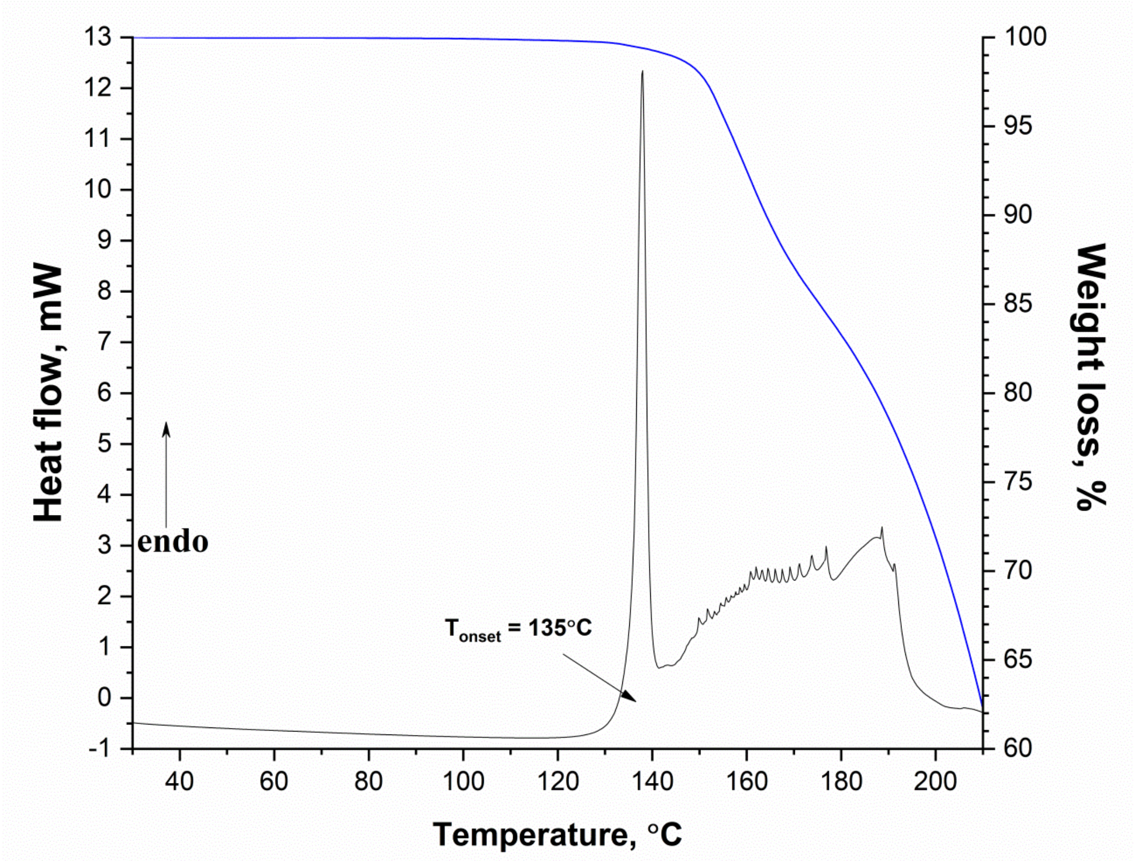

Figure S6. DSC (black line) and TGA thermograms (blue line) of [RLZ+2,4DHBA] recorded at 10 °C min^{-1}.

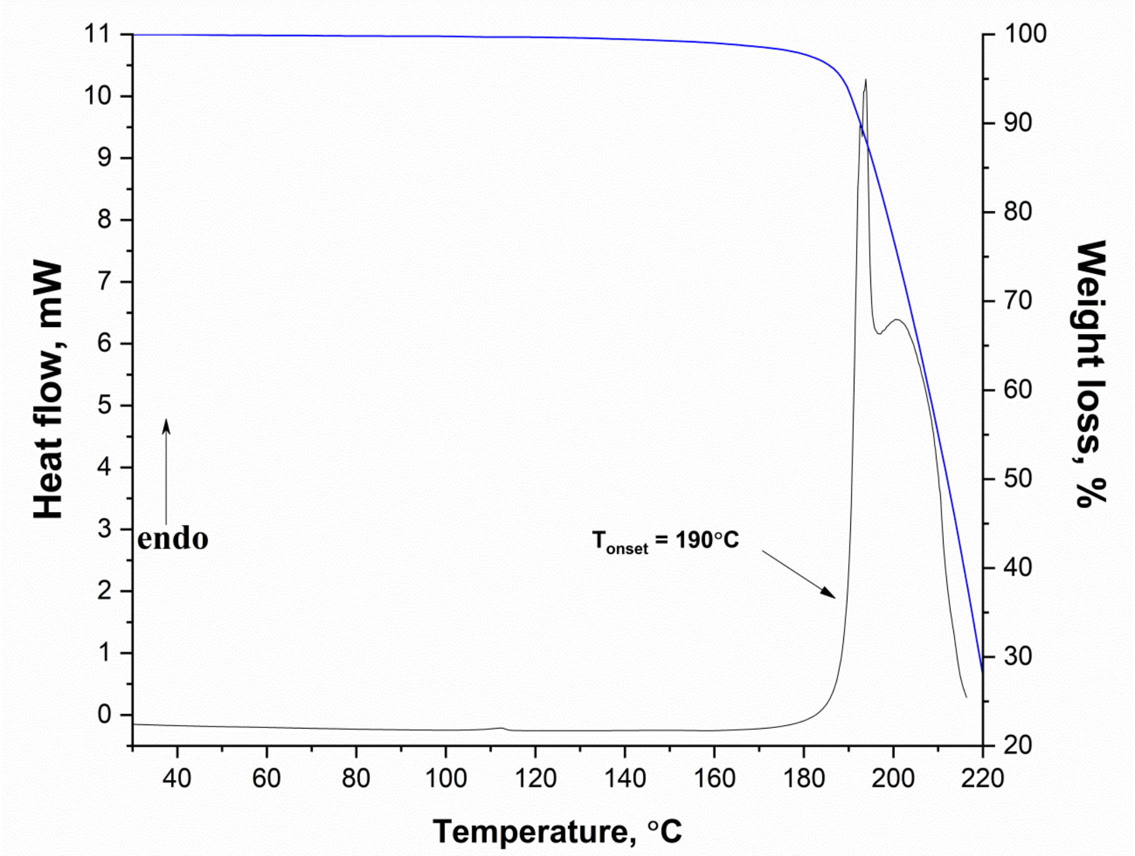

Figure S7. DSC (black line) and TGA thermograms (blue line) of [RLZ+2,6DHBA] recorded at 10 °C min^{-1}.

References

1. Cruz-Cabeza, A.J. Acid–base crystalline complexes and the pKa rule. *CrystEngComm* **2012**, *14*, 6362-6365, doi:10.1039/C2CE26055G.

2. Yadav, B.; Balasubramanian, S.; Chavan, R.B.; Thipparaboina, R.; Naidu, V.G.M.; Shastri, N.R. Hepatoprotective Cocrystals and Salts of Riluzole: Prediction, Synthesis, Solid State Characterization, and Evaluation. *Crystal Growth & Design* **2018**, *18*, 1047-1061, doi:10.1021/acs.cgd.7b01514.

3. Mondal, P.K.; Rao, V.; Mittapalli, S.; Chopra, D. Exploring Solid State Diversity and Solution Characteristics in a Fluorine-Containing Drug Riluzole. *Crystal Growth & Design* **2017**, *17*, 1938-1946, doi:10.1021/acs.cgd.6b01894.

4. Vecchio, S.; Brunetti, B. Thermochemical study of 2,4-, 2,6- and 3,4-dihydroxybenzoic acids in the liquid phase using a TG apparatus. *Thermochimica Acta* **2011**, *515*, 84-90, https://doi.org/10.1016/j.tca.2011.01.001.
